# Clinical diagnosis of pelvic endometriosis: a scoping review

**DOI:** 10.1186/s12905-015-0196-z

**Published:** 2015-05-08

**Authors:** Hedyeh Riazi, Najmeh Tehranian, Saeideh Ziaei, Easa Mohammadi, Ebrahim Hajizadeh, Ali Montazeri

**Affiliations:** Department of Reproductive Health and Midwifery, Faculty of Medical Sciences, Tarbiat Modares University, Jalal-e Al Ahmad Highway, P.O Box 14115-331, Tehran, Iran; Department of Nursing, Faculty of Medical Sciences, Tarbiat Modares University, Jalal-e Al Ahmad Highway, P.O Box 14115–331, Tehran, Iran; Department of Biostatistics, Faculty of Medical Sciences, Tarbiat Modares University, Jalal-e Al Ahmad Highway, P.O Box 14115-331, Tehran, Iran; Mental Health Research Group, Health Metrics Research Center, Iranian Institute for Health Sciences Research, ACECR, P.O Box 13185–1488, Tehran, Iran; Faculty of Humanity Sciences, University of Science & Culture, ACECR, Tehran, Iran

## Abstract

**Background:**

Accurate and timely diagnosis of endometriosis is associated with confusion. Clinical manifestations, imaging techniques, biomarkers and surgical techniques are used as diagnostic approaches. This paper reviews current evidence on clinical manifestation in order to help practitioners and perhaps improve women’s health.

**Methods:**

A review of the literature on clinical diagnosis of pelvic endometriosis that appeared in the English language biomedical journals was performed using PubMed, Science Direct and Google Scholar. The search strategy included the combination of key words ‘endometriosis’ and ‘diagnosis’ or ‘clinical diagnosis’ in the titles or abstracts of articles. The search included all papers published during the year 2000 to 2014. Then, the findings were classified in order to summarize the evidence.

**Results:**

Using the Preferred Reporting Items for Systematic Reviews and Meta-Analyses (PRISMA) statement, in all 51 papers were found relevant and included in this review. In general we found three categories of diagnostic approaches for clinical manifestation including: i) diagnosis via symptoms obtained from history taking, ii) diagnosis via signs obtained from physical examination and iii) diagnosis via risk factors obtained from history taking.

**Conclusion:**

Diagnosis of endometriosis is a matter of concern. Since the disease is associated with diverse clinical symptoms and signs, deeper and more comprehensive consideration according to patient’s history and clinical findings is recommended for early and more accurate detection in order to prioritize women for further investigation and contribute to its early management.

## Background

Endometriosis is a disease with considerable prevalence. It has been estimated to affect 10% to 15% of women of reproductive age [1]. The disease has several impacts in general physical, mental, and social well-being. The annual cost for hospital admission is estimated to be in a total around 54 million euros [2]. Diagnostic delay of endometriosis is a problematic issue [3] and it takes 8 to 11 years to be diagnosed with long and expensive diagnostic methods [2,4]. Thus it is essential to contemplate diagnosis as an important topic. The best method for early diagnosis of endometriosis is still full of unknown issues. Research on the diagnosis of endometriosis currently interfaces with four areas including clinical manifestations, imaging techniques, biomarkers and surgical techniques [5]. Laparoscopy, which is considered as the golden standard in endometriosis diagnosis has considerable risks [5], and other diagnostic methods are presented as inaccurate and with some limitations [6]. Therefore it seems that for developing clinical and non-surgical approaches for early detection of disease, effort is needed to have a better understanding of signs and symptoms of the disease [7].

Although there are several useful overview papers on endometriosis, most published papers are unfocused and usually discuss about all aspects of the disease including pathophysiology, diagnosis and treatment. While there is not any internationally recognized noninvasive method for diagnosis, most presurgical diagnostic methods are clinical judgment based on medical history, symptoms and signs [8]. Thus, this study aimed to review the literature on clinical diagnosis of endometriosis in order to discover the clinical criteria for achieving broader and deeper insight on the topic. Clinical manifestations of endometriosis are very broad and diverse issue with a wide range of changes [9]. Particulalrly here we focus on current research on the history taking and physical examination of endometriosis. It is hoped that this review may help to bring more attention about clinical diagnosis of endometriosis and consequently aid in decreasing diagnostic delay and perhaps contribute to improved women’ health.

## Methods

### Definition

Endometriosis is defined as the presence of endometrial tissue (gland and struma) outside the uterus [10]. Endometriosis has been found in almost all of the tissues and organs of the body [11]. In this study we focused on pelvic endometriosis because pelvic endometriosis is the most common form of the disease, and it mostly affects women during their reproductive life [12].

### Search engines

The search engines included PubMed, Science Direct and Google Scholar using the Preferred Reporting Items for Systematic Reviews and Meta-Analyses (PRISMA) guideline [13].

### Search strategy

The search strategy included the combination of key words ‘endometriosis’ and ‘diagnosis’ or ‘clinical diagnosis’ in the titles or abstracts of articles.

### Inclusion and exclusion criteria

All qualitative and quantitative articles in English language biomedical journals from year 2000 to 2014 on pelvic endometriosis were included. Case reports and articles on non-pelvic endometriosis were excluded. The year 2000 was chosen because the intention was to include more recent literature in this systematic review. The initial search was carried out in January 2013 and updated twice in March 2014 and February 2015.

### Data synthesis

The findings from each idivdual paper on clinical diagnosis were retrieved, and were classified. Then different tables were provided to summriaze and present the findings.

## Results

### Descriptive findings

**Figure 1:**
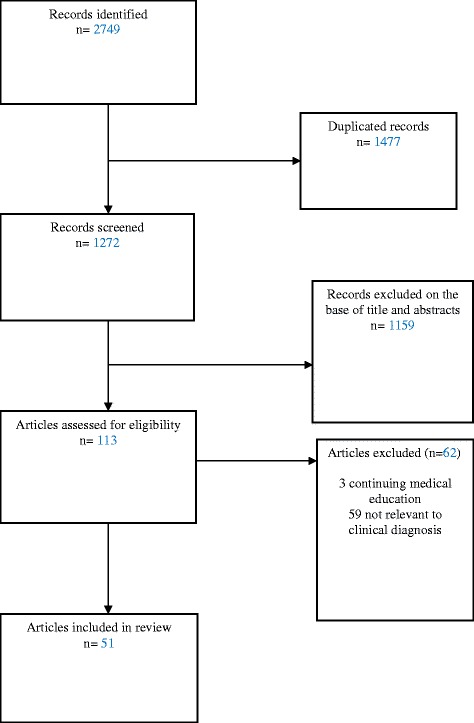
Study selection process.

A total of 2749 citations were identified and after exclusion of duplicates, the abstracts of 1272 citations were screened. Of these, 113 papers were found relevant. However, 51 articles met inclusion criteria and were included in this review. The study selection process is shown in Figure 1. Overall we found 16 overview/commentary publications [2,9,14-27], 33 original research articles [1,7,8,11,12,28-53], two meta-analysis [54,55] and 2 systematic reviews [56,57].

### Review of overview papers

A summary of overview/commentary publications is presented in Table 1. In general, these articles focused mainly on symptoms and treatment of the endometriosis. The emphasis of most papers was on pain, infertility and chronic fatigue.

**Table 1:** **A summary of overview/commentary papers on clinical symptoms and signs**

**Author(s) [Ref.]**	**Year**	**Results for symptoms**	**Results for signs**
Valle [14]	2002	Pelvic pain that often is worse just before and during menstruation, hypermenorrhea, premenstrual staining, dyspareunia, suprapubic pain, dysuria, hematuria, painful defecation (dyschezia), lower back pain.	Local tenderness in cul de sac or uterosacral ligaments, adnexal enlargement or tenderness, pelvic masses.
Spaczynski and Duleba. [15]	2003	Chronic pelvic pain consists of dysmenorrhea, intermenstrual pain, and dyspareunia.	Bluish implants typical of endometriosis or red, hypertrophic lesions bleeding on contact, usually in the posterior fornix. lateral cervical displacement, cervical stenosis. Retroversion, decreased or absent mobility of uterus and tenderness. Tender masses, nodules, and fibrosis appreciated on palpation of the upper vagina, cul-de-sac, uterosacral ligaments, or rectovaginal septum.
Kennedy et al. [16]	2005	Severe dysmenorrhea, deep dyspareunia, chronic pelvic pain, ovulation pain, cyclical or perimenstrual symptoms (e.g. bowel or bladder associated) with or without abnormal bleeding, infertility and chronic fatigue.	Pelvic tenderness, a fixed retroverted uterus, tender uterosacral ligaments or enlarged ovaries on examination. The diagnosis is more certain if deeply infiltrating nodules are found on the uterosacral ligaments or in the pouch of Douglas, and/or visible lesions are seen in the vagina or on the cervix. The findings may, however, be normal.
Mounsey et al. [17]	2006	Pelvic pain, back pain, dyspareunia, dysmenorrhea loin pain, dyschezia, pain with micturition and infertility.	Tender nodules in the posterior vaginal fornix, uterine motion tenderness, a fixed and retroverted uterus, or tender adnexal masses.
Denny and Mann. [18]	2007	Pain around menstruation, dyspareunia, dyschezia, cyclical dysuria and extreme fatigue.	Not discussed.
Amer [19]	2008	Dysmenorrhoea, dyschezia, hematochezia, dysurea, haematurea, dyspareunia, chronic pelvic pain, heavy and/or irregular periods, premenstrual spotting, infertility.	Tenderness on cervical movement, thickening and tenderness of the uterosacral ligaments, fullness or mass in the pouch of Douglas (POD), fixation and retroversion of the uterus, rectovaginal nodule. Adnexal (or even a pelvi-abdominal) mass in women with large endometriomas.
Luisi et al. [2]	2009	Severe dysmenorrhea, deep dyspareunia, chronic pelvic pain, ovulation pain, cyclical or perimenstrual symptoms with or without abnormal bleeding, infertility and chronic fatigue.	Not discussed.
Giudice. [20]	2010	Chronic pelvic pain (lasting ≥6 months), dysmenorrhea, dyspareunia, deep pelvic pain, and lower abdominal pain with or without back and loin pain. The pain can be continuous, and it can be dull, throbbing, or sharp, and exacerbated by physical activity. Bladder- and bowel associated symptoms (nausea, distention, and early satiety) are typically cyclic. Burning or hypersensitivity symptoms that are suggestive of a neuropathic component (infrequently).	A pelvic mass, immobile pelvic organs, and rectovaginal nodules.
Altman and Wolcyzk. [21]	2010	Chronic pelvic pain, dysmenorrhea, dyspareunia, infertility, back pain, dyschezia, rectal pain, diarrhea, constipation, dysuria, hematuria, infertility, chronic fatigue and psychosocial stressors.	Palpable tender nodules in the cul-de-sac or uterosacral ligaments; localized tenderness in the cul-desac, uterosacral ligaments, or rectovaginal septum; pain with uterine movement; enlarged or tender adnexal masses; and fixation of adnexa or uterus in a retroverted position. Red, blue, or hemorrhagic nodules may also be visualized on the external genitalia, vagina, or cervix.
Okeke and Ikeako. [9]	2011	Dysmenorrhea, dyspareunia, menorrhagia and infertility.	Not discussed.
Koninckx et al. [22]	2012	Hypogastric pain, especially dysmenorrhea, deep dyspareunia, severe chronic pain, mictalgia, and dyschezia.	Not discussed.
Acién and Velasco [23]	2013	Dysmenorrhea (during and at the end of menstruation), deep dyspareunia, chronic pelvic pain, and infertility premenstrual spotting for 2–4 days, headache, irritability, or premenstrual tension syndrome.	Not discussed.
Carneiro M M et al. [24]	2013	Dysmenorrhea, dyspareunia, dyschezia, gastrointestinal symptoms, chronic pelvic pain, infertility.	Pelvic tenderness, a fixed retroverted uterus, tender uterosacral ligaments or enlarged ovaries, uterosacral nodularity.
Schrager et al. [25]	2013	Debilitating pelvic pain, dysmenorrhea, dyspareunia, and decreased fertility.	Not discussed.
Mehedintu et al. [26]	2014	Severe dysmenorrhea, non-cyclical chronic pelvic pain, dysfunctional uterine bleeding, infertility, dyspareunia, painful defecation during menstruation, urinary tract symptoms and gastrointestinal symptoms	Not discussed.
Bhattacharjee et al. [27]	2014	Dysmenorrhea, deep dyspareunia, infertility, abnormal uterine bleeding, non-cyclic pain, menstrual cycle abnormalities, constipation, chronic fatigue, heavy or long uncontrollable menstrual periods with small or large blood clots, gastrointestinal problems including diarrhea, bloating and painful defecation, extreme pain in legs and thighs, back pain, mild to extreme pain during intercourse, pain from adhesions which may bind an ovary to the side of the pelvic wall, or they may extend between the bladder and the bowel, uterus, extreme pain with or without the presence of menses, premenstrual spotting, mild to severe fever, headaches, depression, hypoglycemia and anxiety.	Non-specific pelvic tenderness, localized tenderness in the pouch of Douglas, thickened nodular uterosacral ligaments, fixed retroverted uterus, palpable fixed cystic adnexal mass or an obliterated pouch of Douglas, masses, fixity of organs, displacements of cervix & presence of nodules in the rectovaginal pouch or uterosacral ligaments, nodularity or tenderness in the uterosacral ligament, bluish or red powder burn lesions may be seen in the cervix or posterior fornix of the vagina (which may be tender or bleed on touch), bluish nodules in the posterior fornix, a fixed retroverted tender uterus or a firm fixed pouch of Douglas^.^

### Clinical diagnosis

Clinical diagnosis was achieved by different approaches. We have classified these methods in three groups: i) clinical diagnosis by symptoms obtained from history taking, ii) clinical diagnosis by signs obtained from physical examination and iii) clinical diagnosis by risk factors obtained from history taking. These are summarized as follows:

#### Clinical diagnosis by symptoms obtained from history taking

By taking a careful history of patients and considering their symptoms, the disease may be greatly suspected. Many studies recognized symptoms, which were very common such as cyclic or perimenstrual symptoms [16]. A summary of findings on symptoms obtained from history taking are presented here:

##### Pain

Included dysmenorrhea (during and at the end of menstruation) [2,7-9,15-17,19-26,28,29,32-37,42,53,54], pelvic pain before and during menstruation [2,7,14,16-18,21,23,25,33,34], and pain during sexual intercourse or after sex (dyspareunia) [2,7-9,11,15-17,19-25]. Dyspareunia was occurred only with deep thrusting [23]. Lower abdominal pain or suprapubic pain, lower back pain [11,14,17,20,21,29] and loin pain [17,20] were also mentioned. Pelvic pain was present as chronic pelvic pain (lasting ≥6 months) and patients reported that they were suffering from intermenstrual pain (non-period pelvic pain) or ovulation pain [2,7,15,16,19-24,33,34,37,42]. Rectal pain was also mentioned [11,21]. Pain character was found to be throbbing [31,35], dull or sharp and exacerbated by physical activity [20]. Pain often worsened over time and changed in character [20].

##### Menstrual symptoms

Included hypermenorrhea, menorrhagia, premenstrual spotting for 2–4 days [7,9,11,15,22,23,29,32], mid cycle bleeding, irregular bleeding, and irregular periods [2,16,19,26,29].

##### Urinary problems

Included dysuria, hematuria, urinary frequency, urinary tract infection, and cystitis [11,14,17,18,21,23,29,32,34,36,42].

##### Digestive symptoms

Included abdominal bloating, diarrhea with period, painful bowel movements and painful defecation (dyschezia) during menses, hematochezia, nausea and stomach upset at the time of period, constipation, [11,14,16-20,22,24,25,28,29,34,35], irritable bowel syndrome (IBS) [7,32] and early satiety [20].

##### Gynecologic comorbidities

Included gynecological infections and low resistance to infection, candidiasis [11,29,33] infertility [2,7-9,16,17,19,21,23-25,29,34,36,37,42,45], pelvic inflammatory disease, ovarian cysts [7,32,33] and postcoital bleeding [7].

##### Comorbidities

Included a wide range of allergies and allergic disease, dizziness, migraines and headaches at the time of period or before [11,23,29,33,34], and mitral valve prolapse [29].

##### Social life symptoms

Some symptoms such as inability to carry on normal activities including work or school were reported by patients [29]. Depressed and anxious feelings [11], irritability or premenstrual tension syndrome [23], and psychoemotional distress and alexithymia were also mentioned [47].

##### Musculoskeletal symptoms

Included muscle/bone pain, joint pain and leg pain [11].

##### Other symptoms

Patients reported suffering from chronic fatigue, exhaustion, low energy and low-grade fever [2,11,16,18,21,29]. Women also reported burning or hypersensitivity, symptoms that were suggestive of a neuropathic component [20]. Mictalgia was also mentioned [22]. Previous surgery for endometriosis also was found a considerable factor [31,42]. The findings are presented in Table 2.

**Table 2:** **Main diagnostic symptoms and signs obtained from history taking and physical examination respectively**

**Author(s) [Ref.]**	**Year**	**Study design**	**Sample size**	**Findings**
Eskenazi et al. [8]	2001	Prospective study (study sample); retrospective record review (test sample).	90	**Symptoms:** Dysmenorrhea, pelvic pain, dyspareunia and infertility.
				**Signs:** Uterosacral ligament scarring, nodularity, or pain, nodularity or pain in the pouch of Douglas, vaginal endometriotic lesions, painful or fixed adnexal masses, and fixed uterus or pain on movement of uterus in pelvic examination.
Chapron et al. [28]	2002	Retrospective analysis	160	**Signs:** Endometriotic lesions seen in speculum examination and classic, painful, spheric nodule or painful induration in palpation.
Ballweg L M [29]	2004	Cross sectional	7000	**Symptoms:** Fatigue, exhaustion, low energy, numerous gastrointestinal symptoms, abdominal bloating, diarrhea, painful bowel movements or other intestinal upset, nausea and stomach upset at time of period, a wide range of allergies and allergic disease; heavy or irregular bleeding, pain with or after sex, dizziness and headaches at the time of the period and other debilitating symptoms (unable to carry on normal activities, including work or school), in addition to the classic symptom of pain (dysmenorrhea, pain with or after sex (dyspareunia). Lower back pain, pain in the rectum, irregular bleeding, low resistance to infection, infertility, premenstrual spotting, low-grade fever, Pain related to urination, candidiasis, mid cycle bleeding, mitral valve prolapse.
Lemaire. [11]	2004	Descriptive, cross-sectional correlational study.	298	**Symptoms:** Menstrual cramping, fatigue, lower back pain, heavy menstrual flow, non-period pelvic pain, diarrhea with period, allergy, urinary frequency, pain with intercourse, depressed feelings, constipation, muscle/bone pain, headache, anxious feelings, joint pain, leg pain, spotting between/before periods, rectal pain, urinary infection, yeast infection.
Cheewadhanaraks et al. [30]	2004	Prospective study	116	**Signs:** Tenderness and/or nodularity of the cul-de-sac and/or uterosacral ligament(s).
Chapron et al. [31]	2005	Cross sectional	134	**Symptoms:** Painful defecation during menses, severe dyspareunia, pain other than noncyclic, and previous surgery for endometriosis.
Ballard and Mangubat. [32]	2007	National community-based case–control	27715	**Symptoms:** Pelvic pain and dysmenorrhea, dyspareunia, menorrhagia, urinary symptoms (dysuria, cystitis, and urinary tract infections), irritable bowel syndrome (IBS), pelvic inflammatory disease and ovarian cysts.
Flores et al. [33]	2008	Cross sectional	1285	**Symptoms:** Dysmenorrhea, dyspareunia, conceiving problems, chronic pelvic pain, ovarian cysts, migraines, and gynecological infections.
Greene et al. [34]	2009	Cross-sectional	4334	**Symptoms:** Pelvic pain, menstrual pain, ovulatory and nonmenstrual pain, lifetime presence of all three pain types, heavy bleeding, infertility, bowel symptoms, urinary symptoms, pain with urination, nausea/stomach upset or dizziness/headache during menses.
Ballard et al. [35]	2010	Prospective questionnaire-based	185	**Symptoms:** Throbbing pain and dyschezia.
Abbas et al. [7]	2012	Cohort	62,323	**Symptoms:** Dysmenorrhea, dyspareunia, intermenstrual pain, menorrhagia, ovarian cysts, pelvic pain, postcoital bleeding, infertility, irritable bowel syndrome, pelvic inflammatory disease.
Nnoaham et al. [36]	2012	Prospective, observational, two-phase study	1,396	**Symptoms:** Dysmenorrhea, dyspareunia, pelvic pain, Bowel/urinary symptoms, infertility, family history.
Hadisaputra. [37]	2013	Cross sectional	80	**Symptoms:** Infertility, dysmenorrhea, dyspareunia and chronic pelvic pain. **Signs:** Rectovaginal nodule and cervical tenderness.
Cavaggioni et al. [47]	2014	Case- control	80	**Symptoms: M**ood and anxiety disorders, obsessive-compulsive malfunction, depression, alexithymia.
Heitmann et al. [51]	2014	Retrospective cohort study	80	**Symptom:** Premenstrual spotting of ≥ _2 days
Walch et al. [52]	2014	Prospective, controlled clinical trial	102	**Symptom:** Cyclic leg pain
Barbosa et al. [53]	2014	Cross sectional	387	**Symptom:** Dysmenorrhea was the only clinical symptom

#### Clinical diagnosis by signs obtained from physical examination

Clinical signs of the disease that identified by physical examination (pelvic examination by inspection and palpation) included a broad range of signs. External genitalia and the vaginal surface were usually unremarkable [15]. Findings of physical examination are listed as follows:

##### External genitalia

Visible red, blue, or hemorrhagic nodules on the external genitalia [21].

##### Vagina

Visible red, blue, or hemorrhagic nodules on the vagina, and tender masses, nodules, and fibrosis on palpation of the upper vagina [8,15-17,21].

##### Cervix

Visible lesions on the cervix, tenderness on cervical movement, lateral cervical displacement, and cervical stenosis [15,16,19,21,37].

##### Uterus

A fixed (decreased or absent mobility) and retroverted uterus, and uterine motion tenderness in pelvic examination [8,15-17,19,21,24].

##### Adnexa

Tender or fixed adnexal masses resulting from endometriomas, adnexal enlargement, and pelvic masses [8,14,17,19,21].

##### Posterior vaginal fornix

Tender nodules in the posterior vaginal fornix, bluish implants typical of endometriosis or red, hypertrophic lesions bleeding on contact [15,17].

##### Pouch of Douglas

Fullness or mass or nodularity or pain in the pouch of Douglas, local tenderness or palpable tender nodules in cul de sac [8,14,16,19,30].

##### Rectovaginal septum

Tender masses, nodules, and fibrosis of the rectovaginal septum [15,19-21,37].

##### Uterosacral ligament

Thickening, pain or tenderness or nodularity in uterosacral ligament [8,14-16,19,21,24,30].

The most reported signs included changes in uterus, cervix, adnexa and uterosacral ligament palpation. The findings are shown in Table 2.

#### Clinical diagnosis by risk factors obtained from history taking

Although it was reported that the following risk factors alone are not enough, they could potentially direct physicians toward clinical diagnosis of pelvic endometriosis. All risk factors are presented in Table 3.

**Table 3:** **Risk factors or characteristics of endometriosis patients**

**Author(s) [Ref.]**	**Year**	**Study design**	**Sample size**	**Findings**
Kashima et al. [38]	2004	Case–control	623	Familial tendency.
Hediger et al. [39]	2005	Cohort study	48	Taller, thinner and lower body mass index, late maturers (menarche at ≥14 y) and late to initiate sexual activity (≥21 y), less likely to be gravid, parous, and a current smoker.
Flores et al. [33]	2008	Cross-sectional	1285	Longer length of menses, earlier menarche and shorter cycle length.
Parazzini et al. [40]	2008	Case–control	672	More education, lower body mass index, never smoking and null parity.
Sinaii et al. [41]	2008	Cross-sectional	940	Pelvic pain, subfertility.
Yi et al. [1]	2009	Retrospective review of clinical records	481	Lower BMI.
Bazot et al. [42]	2009	Retrospective longitudinal study	92	Infertility, previous surgery for endometriosis, nulliparity, noncyclic chronic pelvic pain, dysmenorrhea, deep dyspareunia, painful defecation, dysuria and asthenia.
Treloar et al. [12]	2010	Case–control	268	Early menarche and early history of dysmenorrhea.
Lafay et al. [43]	2011	Case–control	476	Lower body mass index.
Chapron et al. [44]	2011	Cross-sectional	229	Positive family history, more absenteeism from school during menstruation, OC pill use for treating severe primary dysmenorrhea.
Nnoaham et al. [54]	2012	Systematic review and meta-analysis of case–control studies.	18 articles	Early menarche
Peterson et al. [45]	2013	Cohort	626	Infertility history, dysmenorrhea and pelvic pain.
Parazzini et al. [55]	2013	Metaanalysis	15 articles	Alcohol consumption
Borghese et al. [46]	2014	cross-sectional	663	Rhesus negativity
Xie et al. [48]	2014	prospective cohort study	88 623	Severe teenage acne.
Vercellini et al. [49]	2014	Case–control	771	Blue eye color
Tu et al. [50]	2014	Prospective cohort study	9,585	Prior OCP use in nulliparous women
Bungum et al. [56]	2014	Systematic review	5 articles	Increased risk of allergic disorders (asthma, hay fever/allergic rhinitis of the sinus, eczema, food allergy, allergy to either pollen, dust, trees, paint, grasses, cigarette smoke, perfumes/fragrances, cleaning products, foods or environmental chemicals)
Bonocher et al. [57]	2014	Systematic review	6 articles	Inconclusiveness regarding the benefits of physical exercise as a risk factor

##### Risk factors related to menstrual periods

Included early menarche (before age 11–13) or late menarche (menarche at ≥14 y) [12,15,20,33,39,54], short menstrual cycle (≤27 days) [15,17,20,32,58], longer menstrual flow (≥7 days) [15,30], and spotting before onset of menses [11,15,19,23,29,51]. Late menopause [20], early history of dysmenorrhea [12], obstruction of menstrual outflow for example mullerian anomalies and use of pads and tampons were other risk factors [17,20].

##### Risk factors related to patients’ characteristics

Patients who were taller, thinner and had lower body mass index, red hair [1,15,39,40,43] and dysplastic nevi [15] were found to be more prone to pelvic endometriosis. Higher education was considered as another risk factor [18]. Rh-negativity [46], severe teenage acne [48] and blue eye color [49] were also mentioned as risk factors for pelvic endometriosis.

##### Risk factors related to obstetrics and gynecologic history

These included not having a history of pregnancy or delivery, [15,39-42], late initiation of sexual activity (≥21 y) and never used OCPs [17,39].

##### Risk factors related to family history

Included family history of endometriosis for example mother or sister [15,17,36,38,44]. The relative risk of endometriosis in female siblings was found to be 5.7 [42].

##### Risk factors related to nutrition

Consumption of red meat and trans fats were associated with an increased risk of endometriosis, and eating fruits, green vegetables, and omega-3 long-chain fatty acids were associated with a decreased risk [20].

##### Risk factors related to embryonic factors

Included exposure to diethylstilbestrol in utero and low birth weight [20].

##### Risk factors related to patient’s habits

Consumption of one or more alcoholic drinks per week [17,39,40,55].

##### Risk factors related to race

Asian origin increased the risk of the disease [15].

However, prolonged lactation, multiple pregnancies, the combined oral contraceptive, smoking and exercise are reported as protective factors [20]. The most risk factors were related to menstrual periods, patients’ characteristics and family history.

## Discussion

The emphasis of this review was on several aspects of the clinical diagnosis of pelvic endometriosis. All factors that could have been of any help in clinical diagnosis of the disease were reviewed and classified. These findings divided in three main categories. Moreover this article showed that there were many more symptoms than have traditionally associated with endometriosis.

Usefulness of clinical signs and symptoms in the diagnosis of pelvic endometriosis in women who present with infertility was shown in some studies [17]. In addition, it has been reported that some symptoms had greater predictive value. The most commonly reported symptoms leading to a diagnosis were dysmenorrhea and pelvic pain. Treloar et al. reported that dysmenorrhea was associated with a 2.5-fold increased risk of subsequent endometriosis [12]. Pelvic pain was reported by all patients in a study by Greene et al. [34]. Among the other symptoms leading to a diagnosis, subfertility and an ovarian mass were more commonly reported in stages 3–4, whereas dyspareunia was more common in stages 1–2 [41]. Women with endometriosis were 9 times as likely to report dyspareunia as compared to a control group [32]. In Nnoaham et al. study painful defecation during menstruation and a history of benign ovarian cysts strongly predicted any stages of the disease. The stage III and IV disease was predicted with good accuracy based on symptom-based model. They used several variables in their model including indications for surgery, menstrual history, dysmenorrhea, dyspareunia, pelvic pain, bowel/urinary symptoms, pregnancy/infertility history, personal characteristics and family history [36].

Chapron et al. showed that variables such as painful defecation during menses, severe dyspareunia, pain other than noncyclic, and previous surgery were considered as independent predictors for posterior deep infiltrating endometriosis. They showed a diagnostic model that used two independent predictors: painful defecation during menses and severe dyspareunia. The sensitivity of this model for diagnosing posterior deep infiltrating endometriosis was 74.5% and its specificity was 68.7%. They concluded that standardized evaluation of painful symptoms is useful for screening the disease [31]. Pelvic inflammatory disease and ovarian cysts were 6 and 12 times as likely to be made in women with endometriosis [14].

Although no test provides strong evidence for the presence of endometriosis, the symptom of uterosacral pain had the highest positive likelihood ratio for the diagnosis of endometriosis [20]. In addition to the classic symptom of pain, other symptoms such as fatigue, exhaustion, low energy, gastrointestinal problems, abdominal bloating, and a range of allergic diseases also showed a positive association with endometriosis. These are usually dismissed because they are not widely known. These symptoms are very important. A study by Lemaire showed that the three symptoms with highest total symptom distress were fatigue or weariness, menstrual cramping and nonperiodic pelvic pain [11].

Women with endometriosis were twice as likely to report urinary symptoms such as dysuria, cystitis, and urinary tract infections compared with control group [14]. Sinaii et al. did not report any significant relationship between endometriosis and regular, vigorous exercise, regular use of talc as body powder and cigarette smoking [43], but other studies have found a significant association between smoking and endometriosis [40]. In Hediger study, patients were more likely to be late maturers (menarche at ≥14 y) [39] while in other studies early menarche was often cited as a risk factor for endometriosis [12]. In Parazzini study age at menarche and lifelong type of menstrual cycles were not related to the risk of deep or pelvic and ovarian endometriosis [40]. So it seems that further studies are needed to clarify this relationship.

The risk of the disease raised by advancing age within the reproductive years, peaking among women aged 40–44 years [58]. The findings of the Ballweg’s study indicated that the disease was more severe with an early age of onset and thus it is necessary to pay more attention to girls suffering from menstrual pain [29]. Most studies have shown a significant relationship between endometriosis and length of menstrual cycle, menstrual volume, occurrence of irregular menstrual periods, recent pelvic pain or dyspareunia except one [39] which may be attributed to the small sample size of the study. In the Flores’s study among Puerto Rican women the diagnosis of endometriosis was significantly associated with dysmenorrhea, dyspareunia, and chronic pelvic pain, but not with menstrual cycle characteristics [33]. This was probably due to their study on Hispanic population. In Treloar et al. study no association was found between endometriosis and duration of natural menstruation, heaviness of flow, type of sanitary protection used and history of sexual intercourse during menstruation which may be due to recall bias or small size of control group [12]. Ballard and Mangubat showed that menorrhagia in women with endometriosis was 5 times higher that controls [32].

Lafay et al. showed that patients with the lowest BMI (≤18.5) were at a higher risk of deep infiltrating endometriosis. BMI was significantly lower in deep infiltrating endometriosis and ovarian endometrioma patients but not for the superficial endometriosis patients [43]. In Yi et al. study, women with advanced-stage endometriosis had lower BMI than those with minimal or mild disease, and BMI was significantly associated with disease severity [1]. These findings are in line with other studies [39,40], but in Kennedy study the risk of the disease was increased for women with greater peripheral fat [58]. Borghese et al. showed that Rh-negative women were twice as likely to develop endometriosis. [46]. However, there is controversy about the relationship between blood group and endometriosis [46,59,60] and further investigation is needed to determine the role of blood groups in the development of endometriosis. In a study by Xie et al. it was reported that women who had severe teenage acne had a 20% increased risk of endometriosis. Probable mechanisms for such observation might be due to genetic factors, sex hormones including estrogens and immune malfunction [48]. Blue eye color also was another factor. It is argued that perhaps genetic factors, and vitamin D deficiency due to photo-sensitivity might lead to a relationship between eye color and endometriosis [49]. Several studies have shown that the risk of allergic disease could be increased with endometriosis [56].

In Kashima et al. study the relative risk of endometriosis in female siblings of patients was 5.7 [38], which were consistent with other findings [17]. These findings propose a familial tendency and a genetic factor for endometriosis.

Endometriosis occurs more commonly in middle-aged, upper class, ambitious, white women. This may occur due to greater access to medical care and diagnostic tests such as laparoscopy in these women [58]. Several studies have shown different results about the relationship between physical activity and endometriosis so there is inconclusiveness regarding the benefits of physical exercise as a risk factor for the disease [57].

Two important signs must be considered: deep dyspareunia and nodules in the pouch of Douglas [23]. Findings of physical examination vary significantly with location of endometriotic lesions. They can be seen on speculum examination in only 14% of patients, and a classic, painful, spheric nodule can be palpated in only 43% [28]. In Chapron study a nodule was found in 80% of patients with vaginal endometriosis, this rate dropped to only 35% and 33% in those with deep infiltrating endometriosis of the digestive tract and uterosacral ligaments, respectively [28]. High locations of deep infiltrating endometriosis lesions at the level of uterosacral ligaments, bottom of the pouch of Douglas and upper one-third of the posterior vaginal wall explain why results of routine clinical examination are so poor. Cheewadhanaraks showed a positive predictive value of tenderness and/or nodularity of the cul-de-sac and/or uterosacral ligament(s) in diagnosis of the endometriosis [30]. In Howard study no findings on physical examination, including cervical deviation, cervical tenderness, or paracervical tenderness were predictive for the disease [61] but in Bazot et al. study the sensitivity of physical examination was 73.5% for uterosacral ligament endometriosis, 50% for vaginal endometriosis and 46% for intestinal endometriosis. The accuracy of physical examination is higher during menstruation [42].

Eskenazi et al. reported that ovarian endometriosis, but not nonovarian endometriosis, could be reliably predicted with noninvasive procedures. They used history, pain reports, physical examination and ultrasound for prediction and found that these procedures have moderate success in predicting a surgical diagnosis of endometriosis [8].

Attempts to diagnose women using symptoms, clinical findings or ultrasonography had disappointing findings except, possibly, for ovarian endometriosis in Hediger et al. study [39]. Women with endometriosis experience significantly more gynecological, urological and bowel symptoms than women without endometriosis [32], and that the risk of endometriosis increases in women with endometriosis-related symptoms [7].

## Conclusion

Clinical diagnosis of pelvic endometriosis is difficult. Thus considerable efforts are needed to improve the clinical diagnosis of pelvic endometriosis because it could help to prioritize women for further investigation and contribute to its early diagnosis. However, this review provides integrated findings from the literature containing issues related to signs, symptoms and risk factors. The findings suggest that better diagnosis of pelvic endometriosis need a careful and comprehensive investigation about risk factors during history taking and clinical visits. Perhaps every notes during this period by clinicians and midwifes might lead to timely diagnosis and treatment. Further studies with much more focus on signs and symptoms of the disease are recommended for clarifying the present contradictions.
